# A novel biochar-based composite hydrogel for removing heavy metals in water and alleviating cadmium stress in tobacco seedlings

**DOI:** 10.1038/s41598-023-41946-0

**Published:** 2023-09-20

**Authors:** Fu Du, Liping Liu, Yong Pan, Chuang Wu, Ronghao Wang, Zeyu Zhao, Wenpeng Fan, Hao Song, Youzhi Shi, Jian Wang

**Affiliations:** China Tobacco Hubei Industrial LLC, Wuhan, 430040 China

**Keywords:** Environmental chemistry, Environmental chemistry, Plant stress responses

## Abstract

A novel composite hydrogel (AM/CMC/B) synthesized from peanut shell biochar effectively adsorbs heavy metal Cd in water and reduces its toxicity to tobacco seedlings. The hydrogel, prepared via hydrothermal polymerization using acrylamide (AM), carboxymethyl cellulose (CMC), and peanut shell biochar (B), exhibited a maximum adsorption capacity of 164.83 mg g^−1^ for Cd^2+^ and followed a pseudo-second-order kinetic model. In pot experiments, the application of exogenous AM/CMC/B mitigated the inhibitory effects of Cd-contaminated soil on tobacco seedling growth. Addition of 10 mg kg^−1^ Cd resulted in improved phenotype, root system development, enhanced photosynthetic capacity, stomatal conductance (Gs), stomatal number, and increased antioxidant activity while reducing MDA content and leaf cell death. These findings highlight the potential of AM/CMC/B as an environmentally friendly adsorbent for Cd removal from water and for reducing Cd stress toxicity in tobacco and other plants.

## Introduction

Heavy metal contaminants and metalloids, such as Cd, Pb, Hg, As, and Se, are increasingly problematic for the environment worldwide^1^. Soil heavy metal contamination, in particular, is a global issue^2–4^. Among these heavy metal pollutants, cadmium (Cd) is one of the most common and toxic pollutants in soil^5,6^, which has been identified as a severe threat to human, animal, and plant health^7,8^. Cd enters the environment mainly through industrial processes and phosphate fertilizers, and can accumulate in crops before eventually transferring to the food chain^9^. When consumed through food, Cd can enter human bodies, causing considerable kidney and bone damage^10^. Cd stress primarily affects plant growth as well as physiological and biochemical processes^11^, including inhibiting root system growth^12^, reducing nutrient absorption^13^, and impeding chlorophyll synthesis^14^, which, in turn, hinders photosynthesis. Cd stress also causes inhibitions of antioxidant enzymes, which scavenge ROS^15^. Currently, reducing cadmium pollution in plants is a hot topic in research. In situ stabilization of heavy metals in soils with amendments is regarded as a promising, cost-effective, and environmentally friendly remediation approach^16^.

Hydrogels are cross-linked polymers with a network structure and hydrophilic groups, enabling them to absorb large amounts of water without dissolving^17,18^. Due to their diverse functional groups and three-dimensional network structure, hydrogels have emerged as efficient adsorbents for removing heavy metal ions from wastewater^19,20^. Chemical-based hydrogels have been extensively used for the removal of heavy metal pollutants16. Previous researchers have achieved remarkable results by preparing hydrogels to adsorb pollutants in water. For example, Maciel et al.^21^ created magnetic nanocomposite hydrogels using carrageenan and maghemite, which removed approximately 40% and 30% of each heavy metal contaminant. Qu et al.^22^ demonstrated the effectiveness of hydrogels in removing Pb^2+^, Cd^2+^, and Cu^2+^ from water. Perumal et al.^23^ discovered that chitosan/gelatin hydrogels could remove Cd^2+^, Pb^2+^, Hg^2+^, and Cr^3+^ from water at a removal rate of approximately 73–3.94%. Similar results have been obtained by many researchers^24^. Hydrogels have also shown promise in removing soil pollutants^25^. Liu et al.^26^ developed a ferrous sulfide (FeS) nanoparticle@lignin hydrogel composite, which significantly reduced the total, surfactant-soluble, and fixed Cd in heavily and lightly polluted paddy soils by 22.4–49.6%, 13.5–68.6%, and 40.1–16.6% respectively.

Biochar is a cost-effective solid product derived from organic materials through an oxygen-limited pyrolysis process. These organic materials include agricultural and forestry wastes, such as wood, straw, and fruit shells, as well as organic wastes from industrial and urban life such as garbage and sludge^27^. Biochar has been used as a passivator to immobilize toxic trace elements and to reduce their bioavailability, due to its highly porous structure, surface functional groups, and cation exchange capacity^28^. According to multiple reports, biochar is effective at removing heavy metal pollutants, such as Cd (II) in water and soil^29–33^. Wang et al.^34^ have suggested that the use of biochar amendments could decrease concentrations of arsenic and cadmium in plants, thus reducing the potential risk of metal(loid)s entering the food chain. Additionally, biochar has the potential to increase crop yields and improve crop quality^35^.

Tobacco is an important economic and model crop^36^. However, it is more acclimated to Cd uptake than other crops, readily causing a risk for human health through the inhalation of smoke from cigarettes^37,38^. In view of the advantages of biochar and hydrogel in removing heavy metals in water and soil, this study used the hydrothermal polymerization method to embed biochar into hydrogel to prepare peanut shell biochar-based porous composite hydrogel (AM/CMC/B). Firstly, the adsorption effect of hydrogel on Cd in water was explored through adsorption test, and then its effect on the growth and development of tobacco seedlings under Cd stress was explored through pot experiment. As far as we know, there are few reports on peanut shell biochar-based porous composite hydrogel, and the application of this kind of hydrogel to tobacco to resist Cd stress has not been reported. This study has guiding and reference significance for the development of new heavy metal adsorption materials and plant heavy metal detoxifiers. AM/CMC/B has the advantages of simple synthesis condition and low cost, and is expected to be widely used in the fields of sewage treatment, prevention and control of soil heavy metal pollution and plant resistance to heavy metal stress.

Tobacco is not only a significant economic crop but also serves as a model crop^36^. However, tobacco is more susceptible to Cd uptake than other crops, which puts human health at risk through cigarette smoke inhalation^37,38^. To address this issue, this study aimed to utilize the benefits of biochar and hydrogel in eliminating heavy metals in water and soil. The hydrothermal polymerization method was used to embed biochar into hydrogel to create peanut shell biochar-based porous composite hydrogel (AM/CMC/B). The study first explored the adsorption effect of hydrogel on Cd in water through an adsorption test. Then, it evaluated the effect of this material on the growth and development of tobacco seedlings under Cd stress through pot experiments. To the best of our knowledge, there are limited reports on peanut shell biochar-based porous composite hydrogel, and no studies have explored its application in resisting Cd stress in tobacco. The significance of this study is that it serves as a resource for developing new heavy metal adsorption materials and plant heavy metal detoxifiers. AM/CMC/B has the advantages of simple synthesis conditions and a low cost, which makes it a suitable solution for the fields of sewage treatment, prevention and control of soil heavy metal pollution, and plant resistance to heavy metal stress.

## Materials and methods

### Chemicals

Carboxymethylcellulose sodium (CMC), the degree of polymerization is 800 to 1200, Acrylamide (AM), were supplied by Aladdin Industrial Corporation. *N*, *N*-methylenebisacrylamide (MBA), and ammonia persulfate (APS) were supplied by Sigma–Aldrich. CdN_2_O_6_·4H_2_O waspurchased from Shanghai Yien Chemical Technology Co., Ltd. All chemical reagents were of analytical grade. Deionized water was used in all experiments.

### Preparation of biochar

Biochar (B) was produced under no‑oxygen conditions using peanut shell. Before biochar production, air dried peanut shells were cut into small segments (around 1 cm in length) and then ground and sieved to < 0.3 mm. A crucible sealed with tin foil was filled with the peanut shell, which was then placed in a muffle furnace, heated to 450 °C and held for 4 h before cooling to room temperature. The resulting biochars were then grinded to powder and passed through a 0.15 mm sieve (100 mesh), sealed preservation. All the samples in this experiment were collected from the Tobacco Laboratory of Henan Agricultural University (113.33° E, 34.78° N).

### Preparation of composite hydrogel AM/CMC

**Figure 1:**
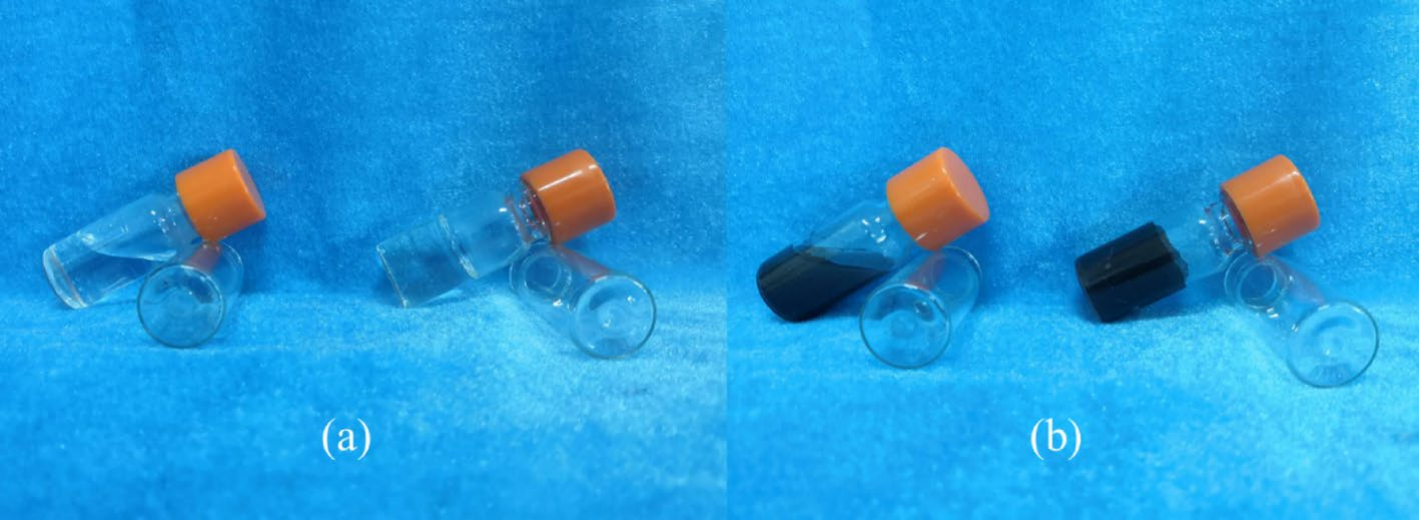
Composite hydrogel AM/CMC (**a**) and composite hydrogel AM/CMC/B (**b**).

1.0 g CMC was placed in the 100 mL beaker and added to 100 mL deionized water, stirring until CMC was completely dissolved. Then 8.0 g AM was added to the beaker at a mass ratio (AM:CMC) of 8:1. After that, added 0.09 g APS and 0.045 g MBA, and stirred well with glass rod, adjusted the rotational speed to 200 rpm and the water bath temperature to 60 °C, reaction time was 3 h. The composite hydrogel AM/CMC (Fig. 1a) was obtained. It can be seen from Fig. 1a that a colorless, transparent and stable hydrogel was formed after the reaction of the mixed solution.

### Preparation of composite hydrogel AM/CMC/B

1.0 g CMC was placed in the 100 mL beaker and added to 100 mL deionized water, stirring until CMC was completely dissolved. Then 8.0 g AM and 4.0 g B were added to the beaker at a mass ratio (AM:CMC:B) of 8:1:4. After that, added 0.09 g APS and 0.045 g MBA, and stirred well with glass rod, adjusted the rotational speed to 200 rpm and the water bath temperature to 60 °C, reaction time was 3 h. The composite hydrogel AM/CMC/B (Fig. 1b) was obtained. It can be seen from Fig. 1b that the mixed solution with added biochar particles formed a black hydrogel after heating.

The prepared hydrogels AM/CMC and AM/CMC/B were swollen by absorbing water in deionized water for 24 h, then they were rinsed three to four times with deionized water. After that, dried them in an oven at 60 °C to a constant weight, crushed by a grinder, passed through a 0.3 mm (60 mesh) pore diameter sieve, sealed with a self-sealing bag and placed in a vacuum dryer.

### Characterization

FTIR refers to the method of Gan et al^39^. Two kinds of hydrogel samples were mixed with KBr and ground into powder to prepare KBr tablets. Fourier transform-infrared (FT-IR) spectroscopy spectra patterns of samples were obtained using a FT-IR spectroscope (Nicolet iS10 FT-IR spectrometer, USA) in the range of 4000–500 cm^−1^. OMNIC infrared spectrum analysis software was used to identify and analyze the characteristic peaks of hydrogel spectra. Two kinds of hydrogel samples were frozen for 4 h, and dried for 20 h in a freeze-dryer, then the surface morphology of them were determined by scanning electron microscopy (SEM) (SIGMA-500, Zeiss, Germany). Prior to observation, the samples were dried in air and sputter-coated with gold.

### Adsorption of Cd^2+^ in solution

The standard solutions of Cd^2+^ ions were prepared by dissolving CdN_2_O_6_·4H_2_O in the deionized water, respectively. The adsorption experiments were executed in plastic tubes containing 100 mL metal ion solutions and 0.05 g dry AM/CMC and AM/CMC/B hydrogels inside. We studied the equilibrium adsorption capacity of the two hydrogels for Cd^2+^ at the fixed temperature (25 °C), pH (2–6), fixed amount of dry hydrogel (0.05 g), fixed initial Cd^2+^ concentrations (200 mg L^−1^) on the adsorption of Cd^2+^. The adsorption experiments of Cd^2+^ on AM/CMC and AM/CMC/B were conducted by 100 mL plastic tubes, which were placed in a constant temperature water bath oscillator with the speed of 150 rpm. After reaching the balance, the concentrations of Cd^2+^ in solutions were measured by a Vista-MPX ICP-OES Inductively coupled Plasma Atomic Emission Spectrometer. At last, the maximum adsorption capacity (Q_m_) of AM/CMC and AM/CMC/B for Cd^2+^ could be calculated by the following equation:

1 $${\text{Q}}_{{\text{m}}} = \, ({\text{C}}_{0} - {\text{C}}_{{\text{e}}} ){\text{V}}/{\text{m}}$$

where the C_0_ and C_e_ are the concentrations (mg L^−1^) of initial and final Cd^2+^ in solutions. V is the volume (L) of Cd^2+^ ionsolutions and m is the weight (g) of the two dry hydrogels.

The adsorption kinetics of Cd^2+^ was investigated in a 100 mg L^−1^ Cd^2+^ solution at 25 °C, pH 5.0. The pseudo-first-order (Eq. 2) and pseudo-second-order kinetic (Eq. 3) models were applied to fit the experimental data on the base of following equations:

2 $${\text{ln}}\left( {{\text{Q}}_{{\text{e}}} {-}{\text{ Q}}_{{\text{t}}} } \right) \, = {\text{lnQ}}_{{\text{e}}} {-}{\text{k}}_{1} {\text{t}}$$

3 $${\text{t}}/{\text{Q}}_{{\text{t}}} = \, 1/{\text{k}}_{2} {\text{Q}}_{{\text{t}}}^{2} + {\text{ t}}/{\text{Q}}_{{\text{e}}}$$

where Q_t_ (mg g^−1^) is the absorption capacity at time t (min), Q_e_ (mg g^−1^) is the equilibrium adsorption capacity, k_1_ (L min^−1^) and k_2_ (g mg^−1^ min^−1^) are the rate constants of the pseudo-first-order model and the pseudo-second-order model, respectively^40^.

### Determination of biomass and relative water content (RWC) of tobacco

After 15 days Cd stress, the tobacco seedings were subjected to biomass. For each measurement, there were kept three replications with different number of plants. The fresh weights of the aboveground and underground portions were taken separately according to the method of Zhang et al.^41^ Fresh tobacco leaves were taken to measure the relative water content (RWC) as per the method of Begun et al.^42^ RWC was calculated with the given formula:

4 $${\text{RWC}}\left( \% \right) \, = \, \left( {{\text{FW}} - {\text{DW}}} \right)/\left( {{\text{TW}} - {\text{DW}}} \right) \, \times \, 100$$

where FW and DW are the fresh leaf weight and the dry weight after drying at 65 °C for 24 h, TW is the saturated weight after soakingin water for 1 h.

### Observation of leaf ultrastructure

Stomatal aperture and stomatal density were detected using an NP 900 polarizing microscope (Nexcope, China) with digital photography. After 15 days of Cd stress, to minimize the effects of other factors, the plants were cultured in light for 3 h so that their stomata were fully open before experimentation. The tobacco seedlings of three treatments were moved outdoors and exposed to high temperature and high light for 10 min, then immediately observed and photographed with a microscope. Three tobacco seedlings were selected for each treatment to take pictures.

### Determination of photosynthetic efficiency and SPAD value

The third true leaves of tobacco treated with Cd for 15 days were selected as the materials. An LI-6400 Portable Photosynthesis Analyzer (LI-COR, USA) was used to measure net photosynthesis (Pn), intercellular carbon dioxide concentration (Ci), transpiration rate (Tr) and stomatal conductance (Gs) as described by More et al.^43^. The measuring time was from 9 a.m. to 11:00 on a sunny day. The light intensity and the CO_2_ concentration in the fixed system were 1000 μmol m^−2^ s^−1^ and 400 cm^3^ m^3^. The SPAD value of tobacco was determined by SPAD 502 PLUS portable chlorophyll meter (KONICA MINOLTA, Japan).

### Evaluation of oxidative stress markers raw data

The physiological parameters were analyzed using the 3rd youngest leaves of three plants/treatment in each replicate. The MDA content was detected through the thiobarbituric acid (TBA) reaction as described by Duan et al.^44^. The accumulation of osmoprotectant proline (PRO), catalase (CAT) and peroxidase (POD) activities were examined using specific detection kits (Beijing Solarbio, Beijing, China), according to the manufacturer’s instructions.

### In vivo localization and quantification of H_2_O_2_ and O_2_^·−^

For in situ detection of hydrogen peroxide (H_2_O_2_), we performed 3,3′-diaminobenzidine (DAB) staining as described by Shi et al.^45^ previously with slight modifications. Immersed leaves were incubated in the light at room temperature for 24 h until brown spots became visible and then 95% ethanol was used until decolorization was complete. For in situ detection of hydrogen peroxide (O_2_^·−^), we performed nitro blue tetrazolium (NBT) staining as described by Luo et al.^37^. Tobacco leaves were stained in NBT solution for 5 h at 30 °C, and 95% ethanol was used until decolorization was complete. After that, the stained leaves were scaned and observed with EPSON V800 scanner.

### Determination of cell death by trypan blue staining

Cell death was measured using the Trypan Blue Assay as described by Islam et al.^46^ previously with slight modifications. For the detection of cell death in leaves, the tobacco leaves treated by Cd for 15 days were excised at the base of leaves with a razor blade and were immersed in trypan blue for 6 h through the cut stems. Then the leaves were incubated in boiling ethanol for 20 min to remove the green background followed by photographing with a scanner (EPSON V800, Japan).

### Statistical analysis

The data were subjected to analysis of variance (ANOVA) in SPSS software version 16.0 (IBM, Chicago, USA). All experiments were carried out in triplicate and expressed as mean value ± standard deviation (mean ± SD). Data were analyzed by using one-way ANOVA to compare between more than two groups and Least Significant Difference mothed (LSD) for pairwise comparisons. Significance of the obtained results was judged at the 5% level. Figures were plotted using Graph Pad Prism 8.0 (GraphPad Software, California, USA) and combind by the software Adobe illustrator (AI) CC 2017 (Adobe, California, USA).

### Statement of legality and compliance

All authors solemnly declare that the planting, collection and measurement of experimental data of all tobacco samples involved in this experiment comply with relevant institutional, national and international standards and legislation. All the authors solemnly declare that all the experimental methods, instrument operation flow and data analysis in this experiment are carried out with reference to the previous research methods, and a few experimental methods are modified. However, we ensure that all experimental methods are carried out in accordance with the relevant guidelines and regulations. All the authors solemnly declare that all tobacco samples collected in this experiment are licensed and authorized by the local public security organs and competent authorities.

## Results and discussion

### FTIR analysis of hydrogels

**Figure 2:**
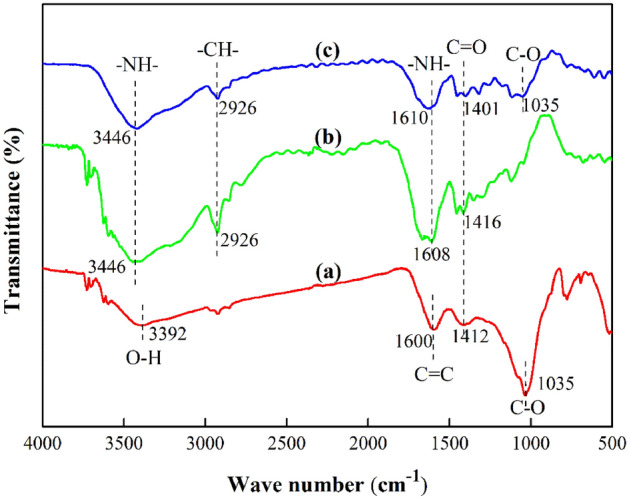
FTIR spectra of B (**a**), AM/CMC (**b**), AM/CMC/B (**c**).

FTIR analysis revealed that the AM/CMC and AM/CMC/B hydrogels (Fig. 2) showed the characteristic functional groups of the AM and CMC units. For biochar, the characteristic peak at 3392 cm^−1^ was due to the bound water and nitrogen hydrogen band and illustrates that some amino groups and hydroxyl groups were present on the surface of the biochar^47,48^. The band at 1600 cm^−1^ corresponded to the stretching vibration C=C of aromatic groups in biochar^49^, and the peak at 1412 cm^−1^ referred to C=O. In addition, there was a significant band at 1035 cm^−1^ corresponding to the stretching C–O of cellulose and hemicelluloses^50^. In the FTIR spectra of AM/CMC hydrogel, the emergence of peaks at 3446 cm^-1^ corresponded to the –N–H stretching and the peak at 2926 cm^−1^ refered to the –CH– stretching vibration. The band appeared at 1608 cm^−1^ (asymmetrical stretching band) was assigned to –NH– bending in the PAM structure^.^ A peak at 1416 cm^−1^ was due to symmetrical stretching vibrations of –COO groups^51^.

As was shown in Fig. 2c, the peaks of AM/CMC/B hydrogel contained the characteristic peaks of B and AM/CMC and the shape of FTIR spectra of AM/CMC/B was the addition of B and AM/CMC spectra. What’s more, the characteristic peak of biochar at 1000 cm^−1^ was obviously weakened. This high sorption ability of AM/CMC/B was connected to the presence of amine (–NH–), and carboxyl (–COO–) groups which possessed strong affinity to metal ions^52^.

### SEM analysis of hydrogels

**Figure 3:**
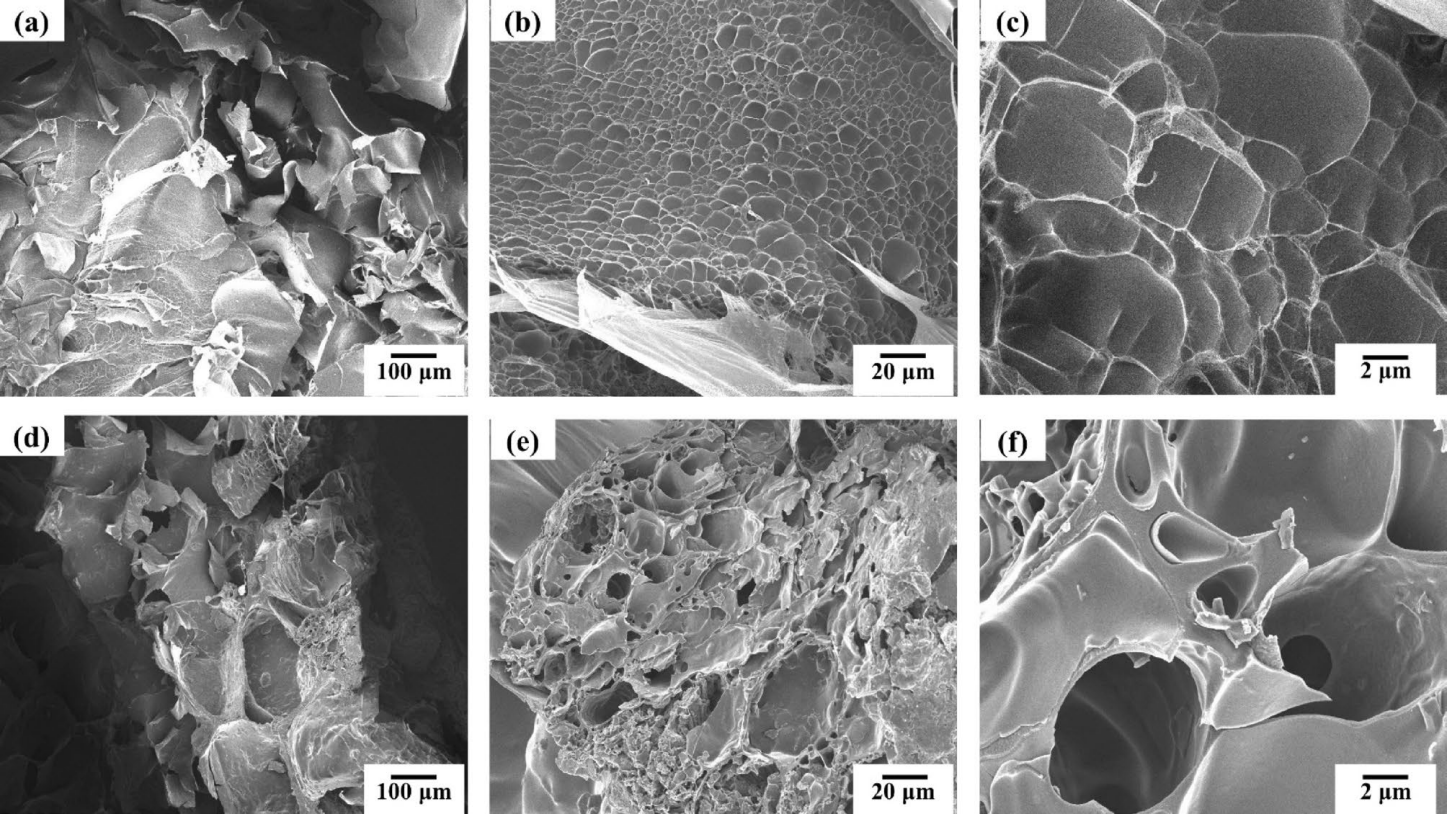
SEM images of AM/CMC (**a**, **b**, **c**) and AM/CMC/B (**d**, **e**, **f**).

The SEM images of samples showing the structural morphology of AM/CMC and AM/CMC/B are represented in Fig. 3a–f. It can be seen from Fig. 3 that a cross-linked network structure are formed inside two hydrogels. As seen in Fig. 3a, the surface of AM/CMC showed a homogenous and smooth creasing pattern. In Fig. 3b,c, we could clearly see that there are a large number of dense, regular and uniform holes in AM/CMC structure which could be attributed to –OH and –COOH hydrophilic functional groups^53^. On the contrary, in Fig. 3d, there was a very rough surface of AM/CMC/B. In addition, there were more holes on the surface than AM/CMC, and the surface of these holes was rougher than AM/CMC, which could be due to the increase of specific surface area of hydrogels by biochar. According to Fig. 3e, f, the biochar particles were scattered on the polymer matrix. What was more interesting was that we could see that many rough holes had been inserted into the holes of AM/CMC/B which were caused by the holes of the added biochar itself. These holes and biochar particles were strong evidence for the adsorption of heavy metals by hydrogel AM/CMC/B^54^.

### Adsorption kinetics and pH of solution

**Figure 4:**
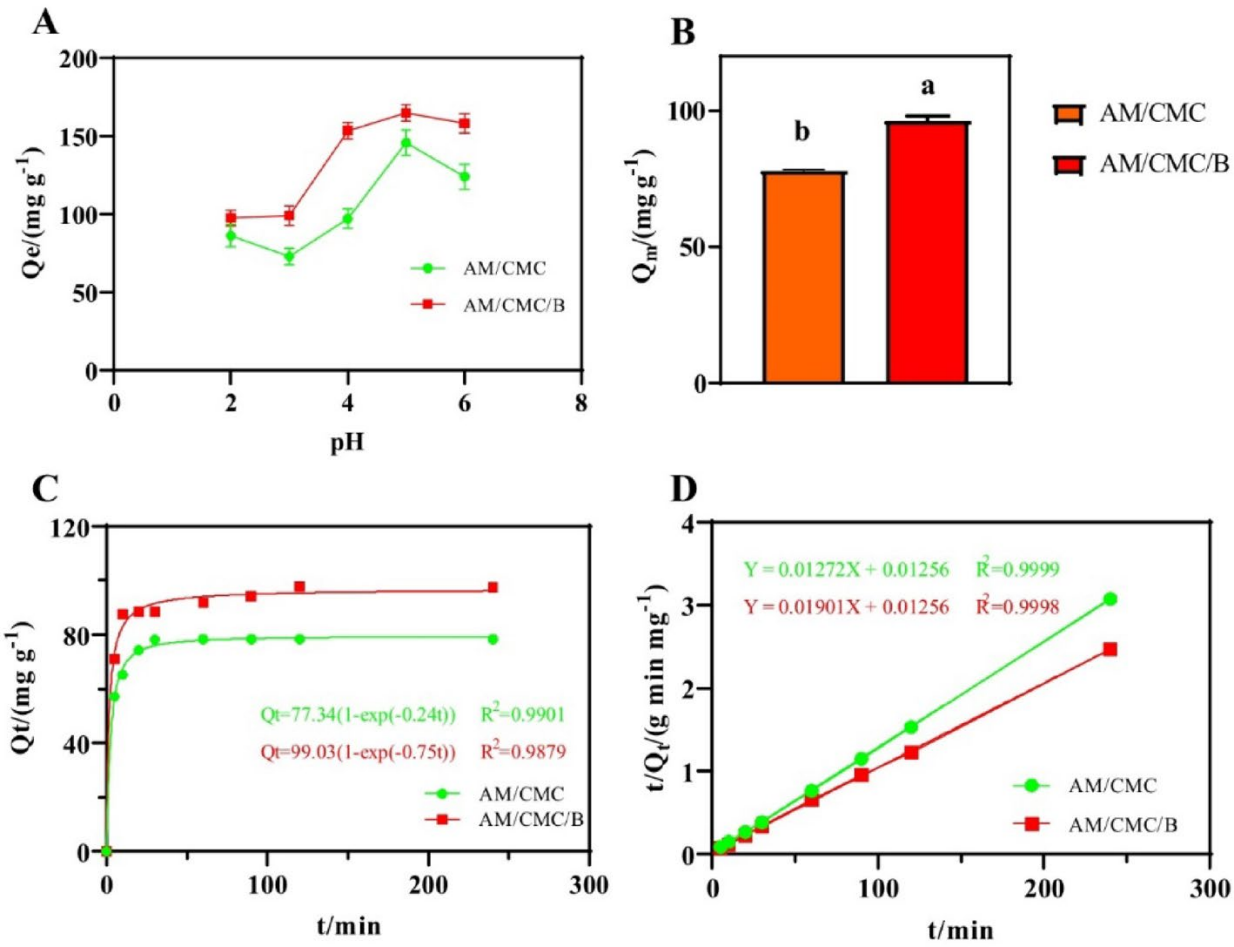
(**A**) Adsorption capacities of Cd^2+^ on AM/CMC and AM/CMC/B at different pH conditions, (**B**) maximum adsorption capacity of Cd^2+^ on AM/CMC and AM/CMC/B, (**C**) pseudo-first-order model of Cd^2+^ by AM/CMC and AM/CMC/B, (**D**) pseudo-second-order model of Cd2^ + ^ by AM/CMC and AM/CMC/B. Different letters mean significantly different between treatments (ANOVA, LSD test, *P* < 0.05).

The initial pH of examined solution is a respectable factor in the removal and recovery of organic and inorganic pollutants from aqueous solution^55^. The effect of pH on the efficiency of AM/CMC and AM/CMC/B for the removal of Cd^2+^ was studied in the 200 mg L^−1^ Cd^2+^ solution at different pH values (Fig. 4A). Generally, the adsorption of metal ion onto the hydrogel composite surfaces follows the ion interaction mechanism which depends on the surface charge of hydrogel composite^49^. As shown in Fig. 4A, when pH was 5, the adsorption capacity of AM/CMC and AM/CMC/B for Cd^2+^ reached the maximum, which was 145.79 mg g^−1^ and 164.83 mg g^−1^, respectively. In Fig. 4B, we could see that in 100 mg L^–1^ Cd^2+^ solution, the maximum adsorption capacity of AM/CMC and AM/CMC/B was 78.51 mg g^−1^ and 96.38 mg g^−1^, respectively. The maximum adsorption capacity of AM/CMC/B for Cd was much higher than that of Li et al.^56^, indicating that the AM/CMC/B synthesized in this study was more competitive for the adsorption of Cd.

As shown in Fig. 4C, adsorption in the first 30 min was found to be very rapid due to the availability of the active sites, while later the rate of adsorption slowed until equilibrium was reached after 4 h. Equation (2) could be transformed into:

5 $${\text{Q}}_{{\text{t}}} = {\text{Q}}_{{\text{e}}} \left( {1 - {\text{e}}_{1}^{{ - {\text{kt}}}} } \right)$$

The Cd^2+^ adsorption behaviors of the two hydrogels were fitted by pseudo-first-order and pseudo-second-order curve models, and the results were shown in Fig. 4C,D. From the fitting results, it could be seen that the adsorption behavior of Cd^2+^ by AM/CMC and AM/CMC/B accords with both pseudo-first-order (R^2^_AM/CMC_ = 0.9901, R^2^_AM/CMC/B_ = 0.9879) and pseudo-second-order (R^2^_AM/CMC_ = 0.9999, R^2^_AM/CMC/B_ = 0.9998) models. What’s more, the maximum adsorption capacity of AM/CMC and AM/CMC/B to Cd^2+^ was 77.34 mg g^-1^ and 99.03 mg g^-1^ respectively, which was very close to the actual measured results of 78.51 mg g^−1^ and 96.38 mg g^−1^. It is obvious that the hydrogel AM/CMC/B had stronger adsorption capacity for Cd^2+^ than AM/CMC, and the adsorption capacity was 17.87 mg g^−1^ higher than that of AM/CMC.

### Effects of hydrogel on phenotype and root system of tobacco

**Figure 5:**
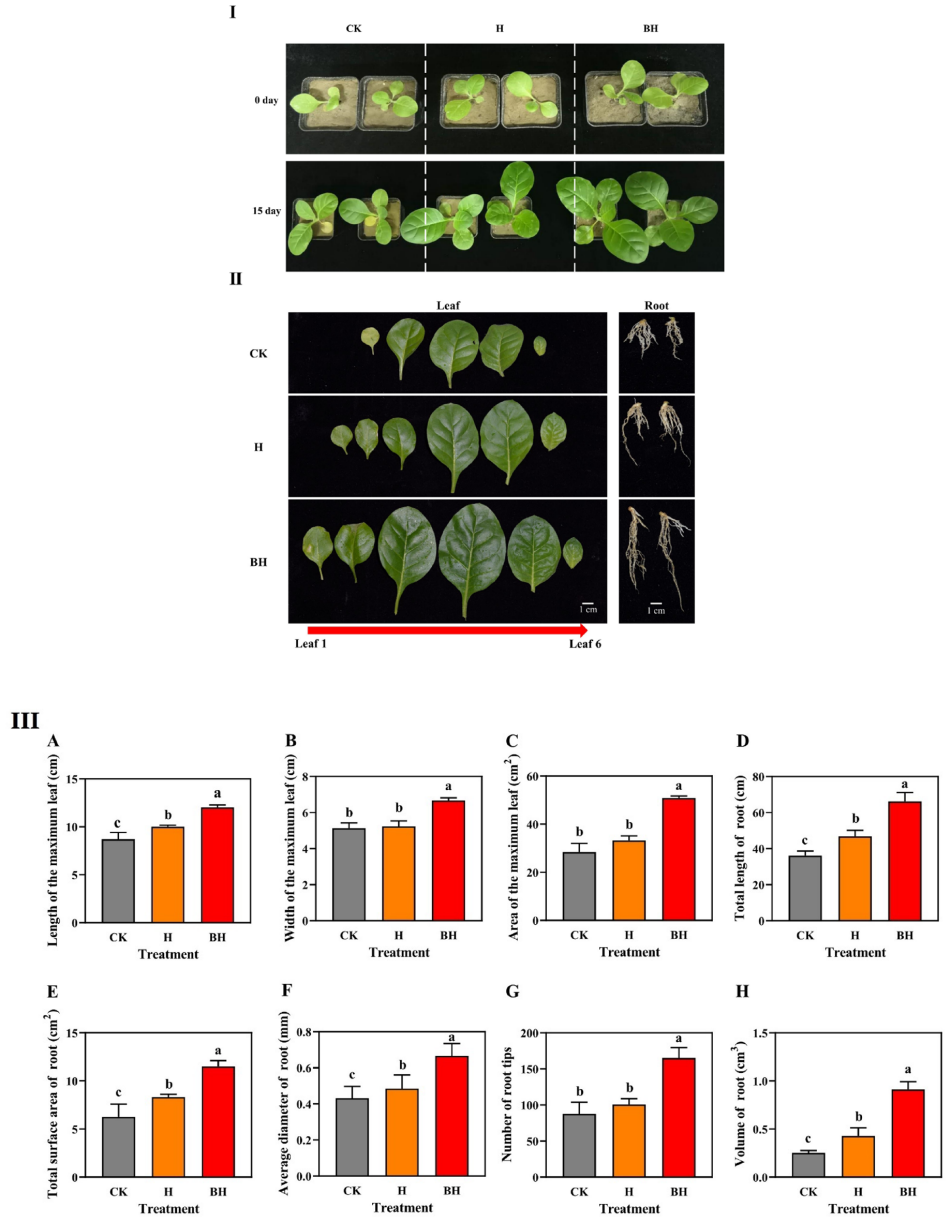
(**I**) Phenotype of tobacco under Cd stress for 0 and 15 days, (**II**) Leaves and roots growth of tobacco under Cd stress for 15 days, (**III**) Growth index of tobacco under Cd stress for 15 days. Different letters mean significantly different between treatments (ANOVA, LSD test, *P* < 0.05).

It could be seen from Fig. 5I that there was no significant difference in the phenotype of tobacco seedlings among the three treatments under Cd stress for 0 day, while there was a significant difference in the performance of tobacco seedlings treated with Cd for 15 days. The growth of tobacco seedlings was BH > H > CK, indicating that tobacco seedlings were most poisoned by Cd without any hydrogels, and their growth and development was the slowest. Roy et al.^57^ had found that leaves was significantly reduced under Cd stress, our study demonstrated similar results. From Fig. 5II, it was showed that the treatments (H and BH) of adding hydrogel increased the total number of leaves of tobacco seedlings and promoted the development of roots under Cd stress. As shown in Fig. 5III, compared with CK, the H and BH treatments all significantly (*P* < 0.05) increased the the maximum leaf length, total root length, total root surface area, average root diameter, number of root tips and root volume under Cd stress. The improvement effect of BH was the greatest. Compared with CK, the H and BH treatments increased the maximum leaf length by 14.94% and 38.28% (*P* < 0.05), the maximum leaf width by 1.94% and 30.01% (*P* < 0.05), the maximum leaf area by 16.90% and 79.19% (*P* < 0.05), and the total root length by 29.74% and 83.31% (*P* < 0.05). The total root surface area increased by 33.00% and 83.97% (*P* < 0.05), the average root diameter increased by 12.36% and 54.44% (*P* < 0.05), the number of root tips increased by 14.83% and 88.21% (*P* < 0.05), and the root volume increased by 69.75% and 261.56% (*P* < 0.05), respectively. These data suggest that H and BH all enabled tobacco seedings to reduce the toxic effect of Cd stress.

The accumulation of Cd in tissues and cells can deleteriously inhibit primary root growth and plant architecture^58^. The above results may be due to the fact that the hydrogel AM/CMC and AM/CMC/B adsorbed Cd, in the process of absorbing water in the soil, which decreased the content of available Cd in the soil, and the toxic effect of Cd on the aboveground and underground parts of tobacco seedlings decreased significantly during the growth process. Therefore, compared with CK, they showed obvious advantages. The most developed root system and the largest leaf area of tobacco seedlings treated with hydrogel AM/CMC/B were related to the maximum adsorption capacity of Cd. Jelusic et al.^59^ obtained similar results using hydrogel.

**Figure 6:**
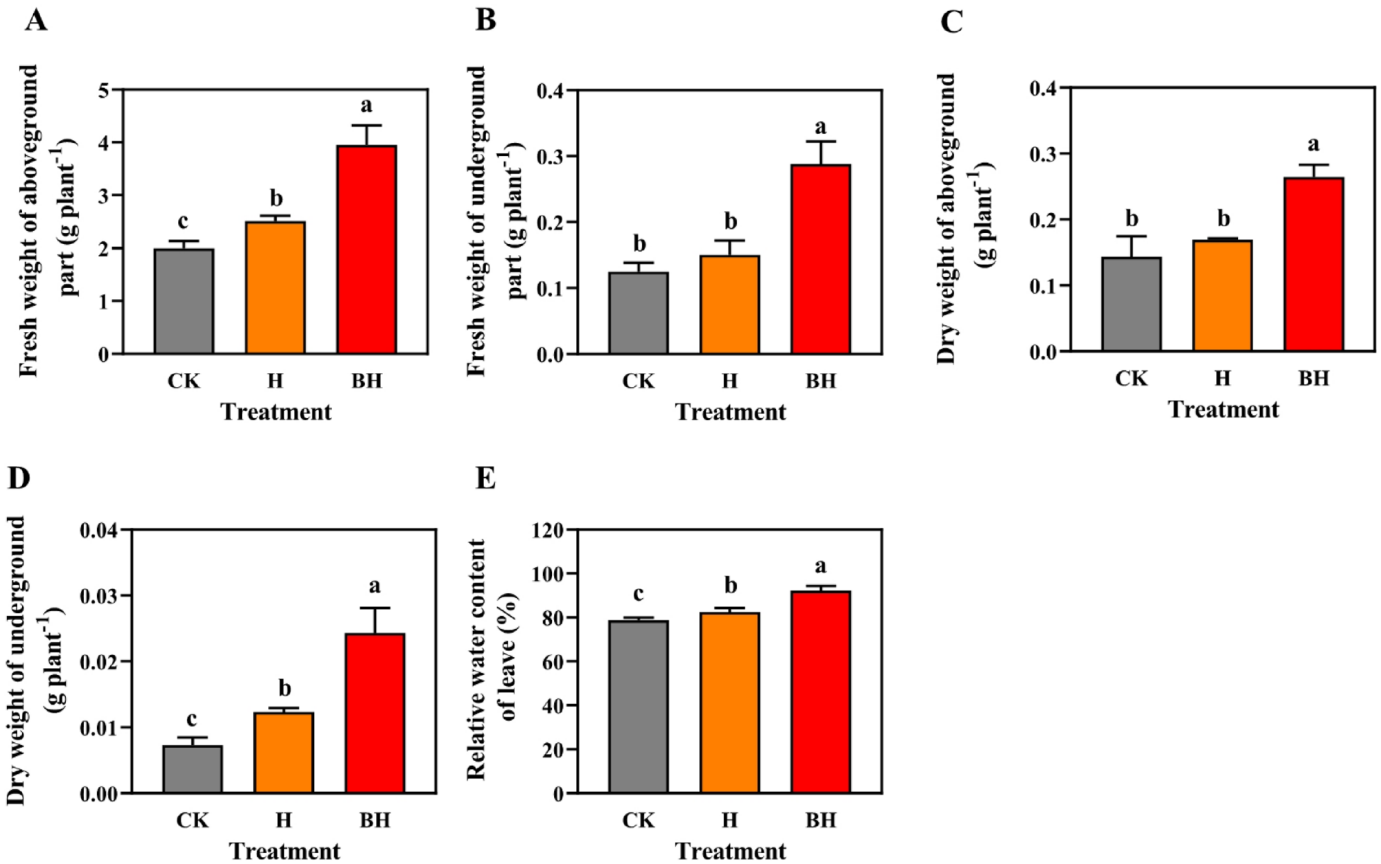
Biomass and relative water content of tobacco seedlings under Cd stress for 15 days. (**A**) Effect on aboveground fresh weight, (**B**) effect on underground fresh weight, (**C**) effect on aboveground plant dry weight, (**D**) Effect on underground plant dry weight, (**E**) effect on leaf RWC. Different letters mean significantly different between treatments (ANOVA, LSD test, *P* < 0.05).

Effects of hydrogel on biomass and RWC of tobacco. It has been reported that the reduction in water absorption rate induced by Cd, causes root and leaf dehydration in plant species^60^. In addition, Cd can significantly reduce plant biomass^61^. These results are consistent with the results of this study. In Fig. 6A–E, the aboveground fresh weight, underground plant dry weight and leaf RWC of H and BH tobacco seedings were significantly higher than those of CK plants (*P* < 0.05). Under Cd stress, the tobacco seedings of CK were most affected by Cd. Compared with CK, the aboveground fresh weight of H and BH treatment increased by 25.91% and 98.39% (*P* < 0.05), the underground fresh weight increased by 20.27% and 130.67% (*P* < 0.05), the aboveground dry weight increased by 18.10% and 84.22% (*P* < 0.05), the underground dry weight increased by 68.18% and 231.82% (*P* < 0.05), and the relative water content of leaves increased by 4.84% and 17.01% (*P* < 0.05). These experimental results showed that hydrogel AM/CMC and AM/CMC/B could alleviate the toxicity of tobacco seedlings under Cd stress and improve their stress resistance. Hydrogel AM/CMC/B was the most favorable to increase the biomass and RWC of tobacco seedlings under Cd stress.

### Effects of hydrogel on leaf stomata of tobacco

Stomata is the unique structure of plant epidermis. Stomata becomes the pathway of air and water vapor in gas metabolism such as carbon assimilation, respiration and transpiration, and its flux is regulated by the opening and closing of guard cells, which is of great physiological significance. Heavy metals perturbate water balance in plants and hence impact stomatal aperture. After longer exposure, stomatal development also is affected, and stomatal density and size can change^62^. Studies had shown that stomata closure was induced directly by heavy metals and/or was a consequence of the early effects of metal toxicity in roots and stems^60^. Previous studies had shown that Cd affected stomata in plant leaves^63^. Cd stress directly affects the density and size of stomata. The stomata number was reliable and useful tool for determining the accumulation level and transport of heavy metal in plant^64^.

**Figure 7:**
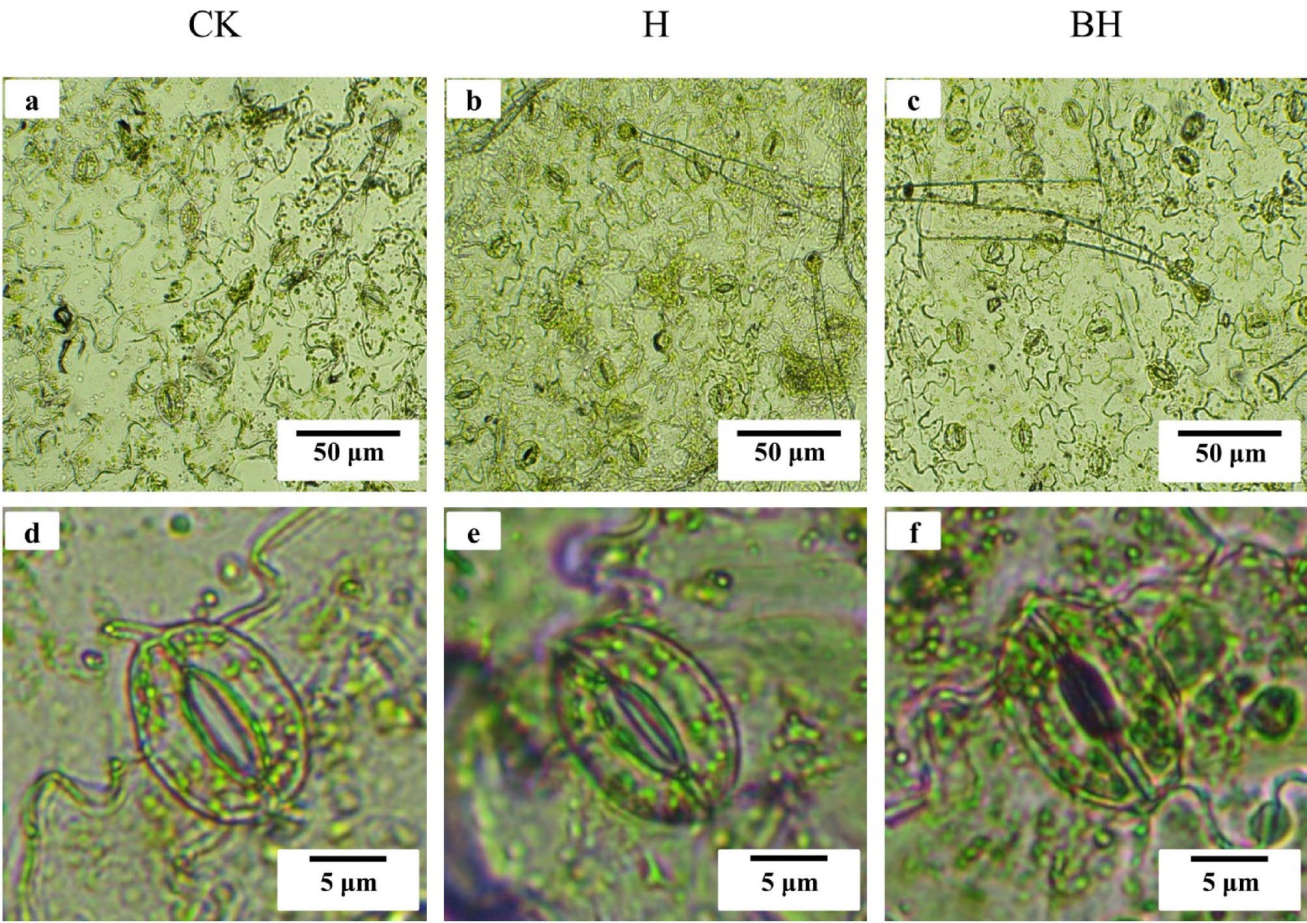
Stomatal morphology of tobacco seedling leaves under Cd stress for 15 days. (**a**, **b**, **c**) The number of stomata in leaves of tobacco seedlings under Cd stress for 15 days, (**d**, **e,** **f**) stomatal size and closure of leaves of tobacco seedlings exposed to high temperature and high light for 10 min after 15 days of Cd stress.

As shown in Fig. 7a–c that the stomata number (density) of leaves of tobacco seedlings treated with H and BH was significantly more than that of CK, and the stomata number of BH treatment was the most, indicating that tobacco seedlings of CK were most poisoned by Cd, and the addition of hydrogel H and BH could alleviate this toxic effect. What’s more, the tobacco seedlings treated with H and BH had more open stomata, and those of BH were the most while the leaf stomata of tobacco seedlings treated with CK basically had no closed stomata. This result was similar to that of Daud et al.^65^.

In Fig. 7d–f, the tobacco seedlings under Cd stress were placed in the sun for 10 min under high temperature and high light stress, and then the stomatal morphology was observed. We found that the stomatal opening degree was CK > H > BH, and the stomata of BH tobacco seedlings were basically completely closed. The reason for this phenomenon might be that plants actively close their stomata under the stress of high temperature and high light, thus reducing water loss. As the tobacco seedlings of CK were most poisoned by Cd, which led to the decline of immune ability, the stress response to external stress was basically lost, and the stomata would not be closed. However, the tobacco seedlings of BH could respond quickly to this stimulus, and the stomata are closed. This result may also be related to the adsorption of Cd in soil by hydrogel AM/CMC/B.

### Effects of hydrogel on photosynthetic efficiency and SPAD value of tobacco

Photosynthesis is an important way for plant growth and dry matter accumulation, it is also one of the processes that are sensitive to adversity, especially heavy metal stress^66^. Sufficient chlorophyll is needed to participate in this process, and SPAD value can directly reflect the amount of chlorophyll in plant leaves. It was reported that Cd stress can inhibit photosynthesis by influencing leaf chloroplast structure, light energy absorption and stomatal conductance^67^. Cd stress can also destroy the chloroplast structure, hinder the synthesis of chlorophyll and reduce the content of chlorophyll^68^.

**Figure 8:**
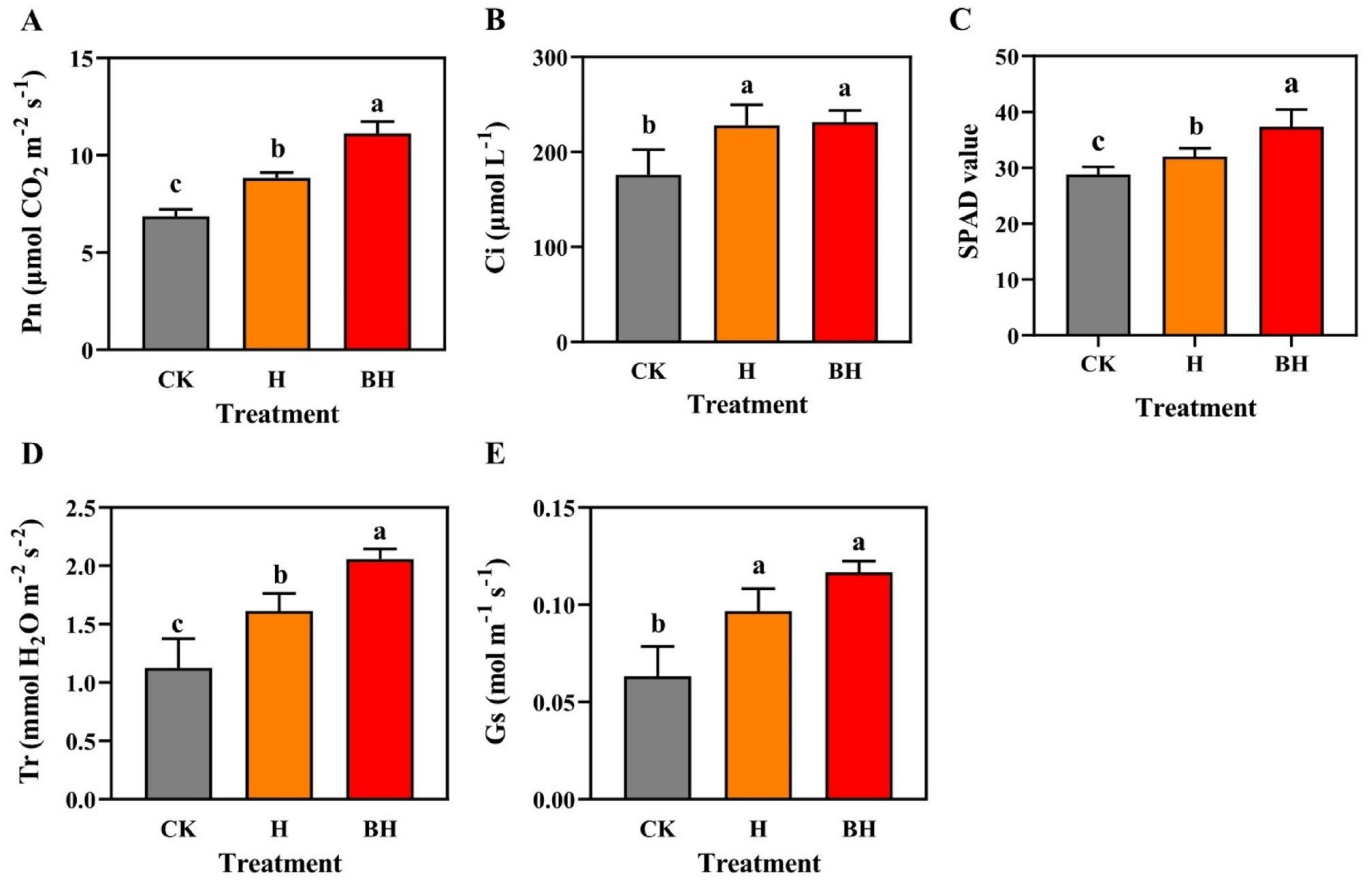
Pn (**A**), Ci (**B**), SPAD (**C**), Tr (**D**) and Gs (**E**) of tobacco seedings leaves under Cd stress for 15 days. Different letters mean significantly different between treatments (ANOVA, LSD test, *P* < 0.05).

From Fig. 8A–E, it could be seen that the Pn, SPAD and Tr of tobacco seedlings treated with H and BH were significantly higher than those of CK (P < 0.05). Compared with CK, the Pn, Ci, SPAD, Tr and Gs of tobacco seedlings treated with B and BH increased by 28.47% and 62.03%, 29.67% and 31.59%, 11.28% and 29.61%, 43.20% and 82.54%, 52.63% and 84.42% (*P* < 0.05). Cd alone stress caused an obvious reduction in SPAD index and Photosynthesis index, whereas application of H and BH alleviated those phenomena. In a word, after 15 days of Cd stress, the inhibition of photosynthesis of tobacco seedlings treated with BH was the least. We speculated that enhanced photosynthetic activity due to BH treatment may be attributed to its adsorption and scavenging ability of Cd in soil.

### Effects of hydrogel on MDA, PRO, CAT and POD contents of tobacco.

**Figure 9:**
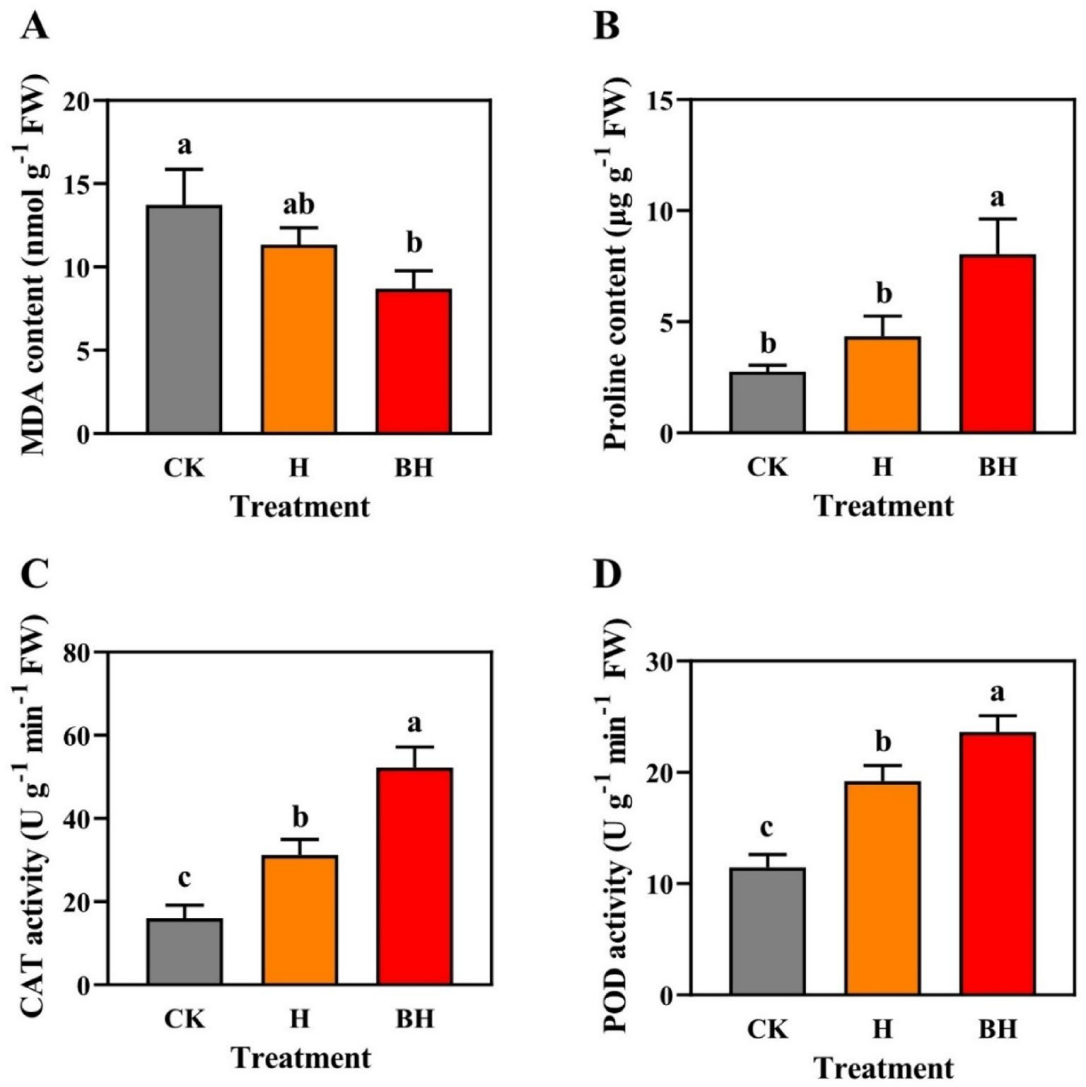
MDA content (**A**), PRO activity (**B**), CAT activity (**C**) and POD activity (**D**) of tobacco seedings leaves under Cd stress for 15 days. Different letters mean significantly different between treatments (ANOVA, LSD test, *P* < 0.05).

Oxidative stress is a central part of abiotic and biotic stresses. This mechanism is caused by a serious cell imbalance between the production of ROS (including H_2_O_2_ and O_2_^·−^) and antioxidative enzymes, which leads to dramatic physiological disorders. The enzymatic ROS scavenging system plays an important role in maintaining the structure and function of membrane and cellular redox equilibrium^69^. For instance, PRO can enhance the antioxidant responses, which finally deal with Cd inhibitory effects^70^. Besides, oxidative damage is closely associated with antioxidative defence machinery. SOD is the first step against ROS generation, converting O_2_^·−^ to H_2_O_2_. Further, H_2_O_2_ can be decomposed to O_2_^·−^ and H_2_O by CAT, thereby decreasing the ROS toxicity^71^. There were significant differences in CAT activity and POD activity among CK, H and BH treatment (*P* < 0.05) (Fig. 9A–D). Compared with CK, the tobacco seedlings treated with BH significantly increased PRO activity, CAT activity and POD activity, and significantly decreased MDA content (*P* < 0.05), the tobacco seedlings treated with H significantly increased CAT activity and POD activity (*P* < 0.05). In more detail, the PRO activity, CAT activity and POD activity of tobacco seedlings treated with B and BH increased by 57.53% and 192.19%, 95.32% and 226.93%, 67.10% and 105.77% (*P* < 0.05), while the MDA content decreased by 17.30% and 36.61% (*P* < 0.05). We thought the above results might be because AM/CMC and AM/CMC/B reduced Cd-induced oxidative damage to an obvious level by strengthening antioxidative response. At the same time, AM/CMC and AM/CMC/B enhanced antioxidant enzyme activities in leaves in response to Cd toxicity, the effect of hydrogel AM/CMC/B was the best.

### DAB, NBT and trypan blue staining of tobacco leaves

**Figure 10:**
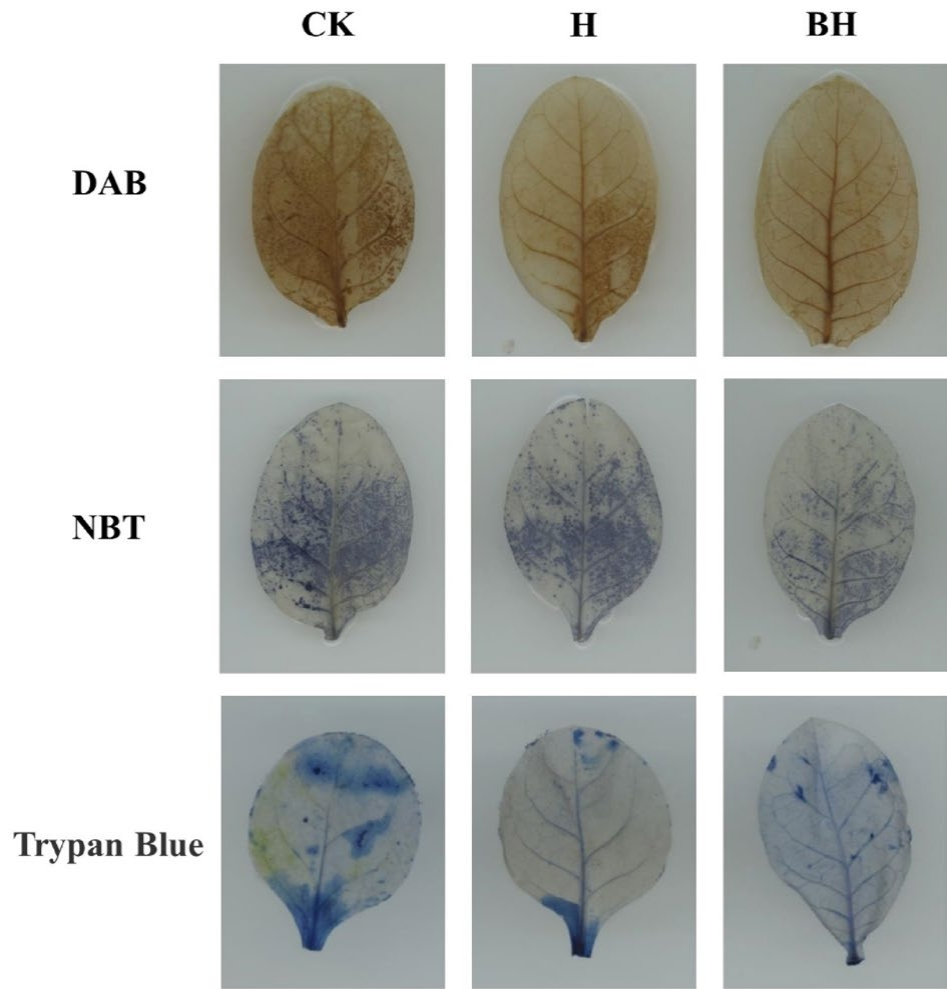
DAB, NBT and Trypan Blue staining of tobacco leaves under Cd stress for 15 days.

Tissue DAB staining showed that after 15 days of Cd stress (showed on Fig. 10), H_2_O_2_ accumulation in tobacco seedlings treated with CK was significant, while H_2_O_2_ accumulation in tobacco seedlings treated with H and BH was much less than that of CK. Tissue NBT staining showed that after 15 days of Cd stress, O_2_^·−^ accumulation was significantly accumulated in tobacco seedlings treated with CK, most of the leaves were dyed blue, and the O_2_^·−^ accumulation of treated H tobacco seedlings was slightly less than that of CK. Meanwhile, the least accumulation was BH treatment, and only a small part of the leaves was dyed blue. The results of Trypan Blue staining showed that the stained area of tobacco seedling leaves treated with CK was the largest and BH was the smallest, indicating that the tobacco seedling cells treated with CK were the most poisoned by Cd, so the number of dead cells was the most. Here we found that BH treatment resulted in the significant decrease in ROS content in tobacco seedlings under Cd stress, which may explain the amelioration of Cd-induced lipid peroxidation and oxidative injury by. Compared with CK, hydrogel AM/CMC/B can effectively reduce the cell death rate under Cd stress, which is most beneficial to the growth of tobacco.

## Conclusion

In this study, peanut shell biochar-based composite hydrogel was successfully synthesized, and its removal effect on heavy metal Cd in water and its mitigation effect on Cd stress in tobacco seedlings were analyzed, which provided a new idea for the prevention and control of heavy metals and the reduction of Cd toxicity. AM/CMC/B can improve the phenotype of tobacco seedlings, increase aboveground and underground biomass of tobacco seedlings, increase the number of leaf stomata, increase photosynthesis and chlorophyll content of tobacco seedlings, reduce the accumulation of ROS under Cd stress, and effectively reduce cell death. The current work showed that the peanut shell biochar-based composite hydrogel had a certain positive effect on alleviating the Cd toxicity suffered by tobacco seedlings. However, the detoxification mechanism of AM/CMC/B on tobacco seedling Cd is still unclear and needs further study.

### Supplementary Information

Supplementary Table S1.

Supplementary Table S2.

Supplementary Table S3.

Supplementary Table S4.

Supplementary Table S5.

## Data Availability

All data generated or analysed during this study are included in this published article [and its supplementary information files].
